# Hydroa vacciniforme as a differential diagnosis of atopic dermatitis

**DOI:** 10.1186/1939-4551-8-S1-A205

**Published:** 2015-04-08

**Authors:** Lucas Brom, Karine Boufleur, João Carlos Simão, Janaina M Melo, Thais Nociti, Ana Maria Roselino, L Karla Arruda

**Affiliations:** 1 Ribeirao Preto Medical School, University of Sao Paulo, Brazil; 2 Clinical Hospital of Ribeirão Preto Medical School, Brazil; 3 Brazilian Association of Allergy and Immunology, Brazil

## Background

Hydroa Vacciniforme is a rare photosensitivity disorder predominantly affecting children. It is characterized by recurrent vesiculopapular eruptions that evolve into necrotic crusts on sun-exposed areas.

## Methods

Case report of a 13-year-old female patient who has been referred to the Allergy Clinic for evaluation of severe Atopic Dermatitis, and had her definitive diagnosis established by skin biopsy.

## Results

Patient’s symptoms started at age 4, presenting with vesiculopapular eruptions and pruritus on photo-exposed areas, evolving into crusts. She had been treated with anti-histamines and topical steroids for several years, with no improvement. Sometimes the patient referred other associated symptoms, including dizziness, dyspnea, chest pain and strong pain in the thighs. Additional treatments included oral corticosteroids, moisturizers, sunscreen filter factor 30 and cyclosporine. On physical exam, the patient presented erythematous papules, infiltrated, associated with lichenification, crusts and chafing on the surface of the exposed areas in the upper limbs, anterior chest, lumbar region and lower limbs, and erythematous plaques in the nasal and malar region. Differential diagnosis included Atopic Dermatitis, Besnier’s Prurigo, Idiopathic Prurigo, Subacute Erythematosus Lupus, Protoporphyria and Contact Dermatitis. At our hospital she underwent skin biopsy, and the results showed characteristic features of Hydroa vacciniforme. The patient now is being treated with chloroquine 250mg/day, Pimecrolimus cream twice daily on the face, mometasone once a week, hydroxyzine 25mg twice daily, and sunblock factor 30. She reports great improvement of symptoms.

## Conclusions

We described a rare case of Hydroa Vacciniforme, demonstrating the importance of this disease in differential diagnosis of Atopic Dermatitis. Correct diagnosis would avoid inappropriate treatment and related adverse side effects.

## Consent

Written informed consent was obtained from the patient for publication of this abstract and any accompanying images. A copy of the written consent is available for review by the Editor of this journal.

